# Development of a Parkinson’s disease specific falls questionnaire

**DOI:** 10.1186/s12877-021-02555-6

**Published:** 2021-10-30

**Authors:** Dale M. Harris, Rachel L. Duckham, Robin M. Daly, Gavin Abbott, Liam Johnson, Timo Rantalainen, Wei-Peng Teo

**Affiliations:** 1 Institute for Physical Activity and Nutrition (IPAN), School of Exercise and Nutrition Sciences, Deakin University, Geelong, Australia; 2 First Year College, Victoria University, Melbourne, Australia; 3 Department of Medicine-Western Health, University of Melbourne, St Albans, VIC 3021 Australia; 4 Australian Institute for Musculoskeletal Science (AIMSS), University of Melbourne and Western Health, St Albans, VIC 3021 Australia; 5 Physiotherapy Department, Epworth HealthCare, Melbourne, Australia; 6 School of Behavioural and Health Sciences, Australian Catholic University, Melbourne, Australia; 7 Faculty of Sport and Health Sciences and Gerontology Research Center, University of Jyväskylä, Jyväskylä, Finland; 8 Physical Education and Sports Science Academic Group, National Institute of Education, Nanyang Technological University, Singapore, Singapore

**Keywords:** Parkinson’s disease, Falls, Near-falls, Reliability, Questionnaire

## Abstract

**Background:**

Falls are a major health burden for older adults with Parkinson’s disease (PD), but there is currently no reliable questionnaire to capture the circumstances and consequences of falls in older adults with PD. This study aimed to develop a PD-specific falls questionnaire and to evaluate its test-retest reliability in older adults with PD.

**Methods:**

A novel PD-specific falls questionnaire (PDF-Q) was developed in two modes (online and paper-based version) and used to assess falls and near-falls events over the past 12-months. Questions were agreed upon by an expert group, with the domains based on previous falls-related questionnaires. The questions included the number and circumstances (activities, location and direction) of falls and near-falls, and consequences (injuries and medical treatment) of falls. The PDF-Q was distributed to 46 older adults with PD (online *n* = 30, paper *n* = 16), who completed the questionnaire twice, 4 weeks apart. Kappa (κ) statistics were used to establish test-retest reliability of the questionnaire items.

**Results:**

Pooled results from both questionnaires for all participants were used to assess the overall test-retest reliability of the questionnaire. Questions assessing the number of falls (*κ* = 0.41) and the number of near-falls (*κ* = 0.51) in the previous 12-months demonstrated weak agreement, while questions on the location of falls (*κ* = 0.89) and near-falls (*κ* = 1.0) demonstrated strong to almost perfect agreement. Questions on the number of indoor (*κ* = 0.86) and outdoor (*κ* = 0.75) falls demonstrated moderate to strong agreement, though questions related to the number of indoor (*κ* = 0.47) and outdoor (*κ* = 0.56) near-falls demonstrated weak agreement. Moderate to strong agreement scores were observed for the most recent fall and near-fall in terms of the direction (indoor fall *κ* = 0.80; outdoor fall *κ* = 0.81; near-fall *κ* = 0.54), activity (indoor fall *κ* = 0.70; outdoor fall *κ* = 0.82; near-fall *κ* = 0.65) and cause (indoor fall *κ* = 0.75; outdoor fall *κ* = 0.62; near-fall *κ* = 0.56).

**Conclusions:**

The new PDF-Q developed in this study was found to be reliable for capturing the circumstances and consequences of recent falls and near-falls in older adults with PD.

**Supplementary Information:**

The online version contains supplementary material available at 10.1186/s12877-021-02555-6.

## Background

Falls are a major health burden for both healthy [1–3] and neurologically impaired [4] community-dwelling older adults as they are often associated with a loss of independence, poorer quality of life and even mortality [5, 6]. It is estimated that between 28 to 36% of healthy community-dwelling individuals aged ≥65 years fall each year [7, 8], but this incidence is markedly higher in older adults with Parkinson’s disease (PD) whereby 35 to 90% are reported to fall at least once per year [9]. Although falls in healthy community-dwelling populations has been extensively explored [10–12], there is less research into the circumstances and consequences of falls for older adults with PD which may differ due to known risk factors that can adversely affect balance and posture, particularly for those with the postural instability and gait disturbance Parkinsonian subtype [13, 14]. Some of the key risk factors include axial rigidity causing poor leaning balance, cognitive impairments, bradykinetic responses to change-in-support strategies, hypokinetic gait and smaller foot clearance upon take-off [6, 15, 16]. Understanding the specific nature of the circumstances and consequences of falls experienced by older adults with PD is therefore important to better inform future falls prevention interventions and to mitigate falls risk factors.

To our knowledge, there are no reliable PD-specific falls questionnaires. To date, most of the previous research examining the circumstances and consequences of falls in older adults with PD have utilised either semi-structured interviews or self-report falls diaries [17–24]. While these methods are the preferred choice when collecting falls information [9, 25], they can be costly, time-consuming, have high attrition rates, and often require continued use of resources (e.g., for data entry and checking, and follow-up phone calls) [25]. In this respect, retrospective analysis using questionnaires to capture falls information may represent a viable alternative, but there are potential limitations such as recall biases [26].

In community-dwelling older adults, questionnaires have been used to assess falls risk factors and falls circumstances [27–30], including one using an online questionnaire to target frail older adults [31], yet the test-retest reliability or validity of these questionnaires has not been reported. In older adults with PD, one study modified a healthy older adult falls questionnaire to capture information on falls [23], but the test-retest reliability of this questionnaire was not performed. Caution is warranted when using questionnaires which are non-PD specific as they may omit key disease-related information such as the use of specific medications, date since diagnosis and/or information related to freezing episodes. Furthermore, many of these questionnaires do not assess or provide an adequate measure of near-falls [17, 32–34], which is important since a history of near-falls is a predictor of future falls in older adults with PD [34]. Therefore, there is a need for a PD-specific questionnaire that can reliably capture falls and near-falls data so that future falls prevention programs can be tailored more precisely towards the needs of older adults with PD.

Although most falls-specific questionnaires have traditionally been administered using the pen and paper method, this method has some limitations including the cost to administer and enter the data from such questionnaires, the time taken for participants to respond and potential limitations related to population reach [35]. As a result, online questionnaires may represent a viable alternative as they can overcome many of these limitations [35, 36]. Two meta-analyses have indicated that both online and paper versions of the same questionnaire yield largely comparable results for self-reported health-related outcomes across both general (e.g. Short Form 36 Health Survey [SF-36]) and disease-specific instruments (e.g., health and quality of life status Parkinson’s Disease Questionnaire [PDQ–39]) [37, 38]. However, some studies have shown subtle differences in the responses between modes, with online questionnaires favouring slightly better health-related outcomes [39–41]. Therefore, this study aimed to develop a PD-specific falls questionnaire, using a step-wise design approach [42, 43], and to test its reliability in two modes (online and paper) among older adults with PD. Test-retest analysis was used to determine the reliability of questions concerning the number and circumstances (e.g., preceding activities, location and direction) underpinning falls and near-falls events, as well as the consequences (e.g., injuries and medical treatment) of falls experienced by older adults with PD over the previous 12-months.

## Methods

### Questionnaire design

Approval for this study was granted by the Deakin University Human Research Ethics Committee (HEAG-H 32_2016). The PD falls questionnaire (PDF-Q) was constructed using a step-wise strategy previously used for the development of reliable questionnaires [42, 43]. This process comprised three phases: (1) development of the questionnaire domains; (2) development of the questions within each domain; and (3) test-retest reliability in a cohort of older adults with PD.

### Phase 1: development of the questionnaire domains

Questionnaire domains were developed based on previous studies exploring falls circumstances among geriatric populations [12, 27, 44], and were subsequently discussed by an expert team of researchers with experience in PD and falls research. This process has been recommended previously to establish content validity of an instrument [42, 43, 45]. A consensus was reached via group discussion regarding the four primary domains of the questionnaire, which were identified as most important when understanding falls experienced by older adults with PD: 1) falls and near-falls history; 2) circumstances of falls; 3) circumstances of near-falls, and 4) consequences of falls. A PD-relevant domain was also designed to include questions on medication, date since diagnosis and freezing episodes. Finally, descriptive questions on general anthropometric (e.g., height and weight) and personal information (e.g., date of birth) were added as general information domain. Both the PD-relevant and the general information domains were added to provide the opportunity for further analyses on potential correlates of falls/near-falls experienced by older adults with PD.

### Phase 2: development of the questions within each domain

From the studies exploring falls circumstances among geriatric populations [12, 27, 44], as well as one study for older adults with PD [22], an initial repository of falls questions was compiled that were most pertinent to capturing falls information from older adults with PD. These studies either listed specific questions within their methodology or referenced a particular questionnaire that was used to capture fall information (e.g., the New South Wales Falls Prevention Baseline Survey [27]). The questions were then narrowed down if they addressed the prevalence or incidence, circumstances and consequences of falls, and were appropriately modified for inclusion in the PDF-Q under each of the relevant domains. In addition, to maintain internal consistency of the instrument, the ‘circumstance of falls and near-falls’ questions were re-adapted based on the recommendations from Stack and Ashburn [24]. All falls-related questions were delineated by location (e.g., indoor or outdoor falls) to capture specific information on how and where falls occurred. Questions to capture information about near-falls were also added, with a clear definition provided to define a near-fall (e.g., “any incident where you may have fallen but were able to catch yourself and did not come to rest on the ground”) [34]. As older adults with PD fall multiple times per year [5, 9, 24], we decided to capture details on the circumstances surrounding the four most recent falls and near-falls events only to avoid potential problems with recall [46]. Once a bank of questions was established, each question within each domain was reviewed, revised, and finalised via consensus by the same expert team of researchers for inclusion within the PDF-Q. Before conducting test-retest analysis, the PDF-Q was pilot tested by three individuals (all male) with PD (mean ± SD, age 70.2 ± 4.6 years; mild-to-moderate PD [Hoehn and Yahr score 2–3]), who were previously enrolled in a clinical exercise trial at Deakin University [47], and who all had experienced a fall(s) in the previous 12-months. These individuals were asked to complete the questionnaire and provide feedback on the domains and content of the questions. Specifically, we asked for feedback on their general understanding of what was being asked for each question, the flow and layout of the questionnaire, and if any difficulties were found in understanding or answering the questions. This end-user feedback was used to modify and refine the PDF-Q before conducting the test-retest evaluation using both a paper and online version of the questionnaire. The final version of the PDF-Q contained six domains: the first domain consisted of general information (e.g., age, consent and person completing the questionnaire) and anthropometric questions, the second domain consisted of disease status questions, and the remaining four domains consisted of falls and near-falls questions (Additional file 1).

### Phase 3: establishing test-retest reliability

Participants were included in this study if they were medically diagnosed with having PD, taking PD medication, and not receiving deep brain stimulation. Participants were excluded if they had not experienced a fall or near-fall in the previous 12-months. Furthermore, participants were asked to complete the Montreal Cognitive Assessment and a cut-off score < 26 of 30 was used to indicate the presence of mild cognitive impairment, which precluded participation in this study.

This study implemented a two-part recruitment process for the online and paper-based questionnaires, which began after pilot testing. To assess test-retest reliability of either the online or paper-based questionnaires, participants were asked to complete the PDF-Q twice, four-weeks apart. A four-week washout period is recommended as an acceptable time period to avoid memory response bias [48]. The time to complete either questionnaire was ~ 20 min. For the online questionnaire, participants were initially recruited from a local community support group where a member of the research team (DMH) conducted a short presentation about the project. From this community support group, 44 members expressed an interest in participating in the study and their contact details were recorded. After a follow up phone call, 30 members met the inclusion criteria and consented to participate and were subsequently emailed an e-link directing them to the online PDF-Q questionnaire to complete via SurveyMonkey™ (Palo Alto, CA 94301, USA, http://www.SurveyMonkey.com), with instructions on when to complete the test and retest questionnaire. For the paper-based questionnaire, 36 older adults with PD were contacted who had previously participated in a research project [35] conducted by our team and had expressed an interest in participating in future studies. From these participants, 16 met the inclusion criteria and consented to participate, and were subsequently asked to complete the paper-based PDF-Q (test and re-test) on-site at Deakin University. Overall, all 46 participants completing the online (*n* = 30) or paper-based (*n* = 16) questionnaire responded to both the test and retest questionnaire. While most responses were self-completed by the participants (online and paper *n* = 41 [89%]; online *n* = 25 [83%], paper *n* = 16 [100%]), some participants completing the online questionnaire submitted responses via proxy (e.g., personal carer) owing to an inability to complete the questionnaire themselves due to typing difficulties (*n* = 5 [11%]). In this regard, the proxy respondents were instructed to list the falls responses based on the information provided directly by the participant only and were required to do the same at retest.

### Statistical analysis

Statistical analysis was conducted using Stata/SE 15 (STATA, College Station, TX, USA). Independent t-test for continuous variables and chi-square tests for categorical variables were used to compare demographic, physical and disease status characteristics between participants that completed the paper and online version of the questionnaire. Test-retest reliability for the PDF-Q was assessed by comparing the agreement between the first and second administered questionnaires for the online and paper-based questionnaire separately and both combined. Levels of agreement were determined using the Kappa (κ) statistic (with standard error) for each fall and near-fall individual questions within the PDF-Q. For ordinal response variables, weighted κ was calculated using the default weighting matrix provided in Stata/SE 15. To provide an estimate of the reliability of each domain, the individual κ scores from each question within each domain were averaged to provide an overall κ score of the domain. The first 10 questions of the PDF-Q consisted of standard physical characteristic and disease-specific items, which did not relate specifically to falls or near-falls and were therefore excluded from the test-retest examination to eliminate a false test-retest reliability outcome. All participants were instructed to respond to all questions. However, for the test-retest analysis, any responses of *“do not remember”*, or unanswered questions, were subsequently coded as missing. The following standards were used to interpret the level of agreement for the κ coefficient between items: 0–0.20 = None, 0.21–0.39 = Minimal, 0.40–0.59 = Weak, 0.60–0.79 = Moderate, 0.80–0.90 = Strong, > 0.90 = Almost Perfect [49].

### Sample size

Previous research suggests that sample sizes of at least 25 participants for test-retest reliability experiments are needed to meet the confidence interval bound ratio requirements [46]. It has also been recommended that sample sizes of ≥30 are needed to demonstrate adequate precision of the estimate of agreement of κ [49]. Due to the small number of participants that we were able to recruit to complete the paper-based questionnaire, and to demonstrate adequate precision of the estimate of agreement of κ, the focus of the results will be on the test-retest reliability outcomes of the pooled (*n* = 46) responses (combined online and paper-based questionnaire data). However, for transparency and descriptive purposes, we have reported the findings from each questionnaire separately.

## Results

Characteristics of participants completing the paper and online versions of the questionnaire and all participants combined are reported in Table 1. On average, participants were aged 71 years and were a mean 9.3 years since formal PD diagnosis. No statistical differences were observed between participants completing the paper and online version of the questionnaire.

**Table 1:** Demographic and physical characteristics, and disease status of older adults with PD

	Online (***n*** = 30)	Paper (***n*** = 16)	Combined (***n*** = 46)
**Questionnaire Details**			
The person who completed the survey			
Self-completed, n (%)	25 (83%)	16 (100%)	41 (89%)
Carer, n (%)	5 (17%)	0 (0%)	5 (11%)
**Physical Characteristics**			
Age (years)	68.6 ± 6.7	73. 3 ± 3.7	71.2 ± 6.1
Height (cm)	166.2 ± 3.6	169.1 ± 2.6	168.1 ± 2.9
Weight (kg)	67.8 ± 5.6	65.5 ± 4.9	67.1 ± 4.8
Body mass index (kg/m^2^)	24.6 ± 3.4	22.9 ± 4.1	23.7 ± 3.5
Sex, n (% male)^a^	20 (67%)	10 (63%)	30 (65%)
**Parkinson’s disease status**			
Years since formal diagnosis	10.2 ± 2.6	8.3 ± 2.2	9.3 (2.4)
Taking medication for PD, n (% yes)	30 (100%)	16 (100%)	46 (100%)

All data are means ± standard deviation (SD) unless otherwise stated (i.e. number [percentage])

*PD* Parkinson’s disease

^a^Sixteen participants did not disclose information on gender

Descriptive statistics and test-retest agreement for each question of the PDF-Q are provided for falls (Table 2), near-falls (Table 3) and injurious falls (Table 4) with the findings for online, paper and combined versions of the questionnaires presented. As indicated previously, due to the small number of participants that completed the paper-based questionnaire (*n* = 16), the focus of the results is on the test-retest reliability outcomes of the pooled responses from both questionnaires combined (*n* = 46). However, for the majority of the individual questions, there was comparable test-retest reliability for the online and paper-based questionnaire. Furthermore, only data for the participants’ two most recent falls and near-falls were included as most participants either could not remember the details of the events beyond the two most recent falls and near-falls (*n* = 29, 63%) or did not have more than two falls or near-falls (*n* = 12, 26%).

**Table 2:** Responses and test-retest reliability for the falls circumstances questions, including the location and direction off the fall, the activity preceding the fall and how the fall occurred

Falls Questions	Online Version (*n* = 30)				Paper Version (*n* = 16)				Combined Versions (*n* = 46)			
	Test	Retest	Test-retest reliability		Test	Retest	Test-retest reliability		Test	Retest	Test-retest reliability	
			Kappa	SE			Kappa	SE			Kappa	SE
Have you experienced a fall in the past 12-months? n (%)	30 (100%)	30 (100%)	–	–	16 (100%)	16 (100%)	–	–	46 (100%)	46 (100%)	–	–
Number of falls in the past 12-months; median (IQR)	2 (1–2)	1 (1–2)	0.47	0.13	2 (1–3)	1 (1–2)	0.30	0.17	2 (1–2)	1 (1–2)	0.41	0.10
Location of falls in the past 12-months; n (%)												
Indoor	21 (70%)	24 (80%)	0.68	0.16	14 (88%)	14 (88%)	1.0	0.25	35 (76%)	38 (83%)	0.91	0.13
Outdoor	8 (27%)	6 (20%)			2 (12%)	2 (12%)			10 (22%)	8 (17%)		
Both	1 (3%)	0 (0%)			0 (0%)	0 (0%)			1 (2%)	0 (0%)		
Number of indoor falls in the past 12-months; median (IQR)	2 (1–3)	2 (1–3)	0.91	0.15	2 (1–3)	2 (1–3)	0.79	0.18	2 (1–3)	2 (1–3)	0.86	0.11
Number of outdoor falls in the past 12-months; median (IQR)	2 (1–2)	1 (1–1)	0.57	0.29	2 (1–2)	1 (1–1)	–	–	1.5 (1–2)	1 (1–1)	0.75	0.13
Most recent indoor falls circumstances ^a^	*n* = 22	*n* = 24			*n* = 14	*n* = 14			*n* = 36	*n* = 38		
*Fall 1: Location of fall*												
Kitchen	8 (36%)	10 (42%)	0.72	0.12	4 (29%)	5 (36%)	0.74	0.15	12 (33%)	15 (39%)	0.74	0.09
Hallway	4 (18%)	5 (21%)			5 (36%)	3 (21%)			9 (25%)	8 (21%)		
Living Room	3 (14%)	2 (8%)			1 (7%)	3 (21%)			4 (11%)	5 (13%)		
Bedroom	3 (14%)	1 (4%)			1 (7%)	1 (7%)			4 (11%)	2 (5%)		
Bathroom	2 (9%)	3 (12%)			3 (21%)	2 (14%)			5 (14%)	5 (13%)		
In another person’s home	2 (9%)	3 (12%)			0 (0%)	0 (0%)			2 (6%)	3 (8%)		
*Fall 2: Location of fall*												
Kitchen	5 (23%)	5 (21%)	0.87	0.11	2 (14%)	1 (7%)	0.91	0.16	7 (19%)	6 (16%)	0.89	0.09
Hallway	10 (46%)	10 (42%)			6 (43%)	7 (50%)			16 (44%)	17 (45%)		
Living Room	7 (31%)	9 (37%)			3 (21%)	4 (29%)			10 (28%)	13 (34%)		
Bedroom	0 (0%)	0 (0%)			3 (21%)	2 (14%)			3 (8%)	2 (5%)		
Bathroom	0 (0%)	0 (0%)			0 (0%)	0 (0%)			0 (0%)	0 (0%)		
In another person’s home	0 (0%)	0 (0%)			0 (0%)	0 (0%)			0 (0%)	0 (0%)		
*Fall 1: Activity during fall*												
Housework	7 (32%)	6 (25%)	0.60	0.12	6 (43%)	7 (50%)	0.91	0.19	13 (36%)	13 (34%)	0.70	0.09
Getting out of bed	9 (41%)	12 (50%)			6 (43%)	6 (43%)			15 (42%)	19 (50%)		
Getting in/out of shower	2 (9%)	2 (8%)			2 (14%)	1 (7%)			4 (11%)	3 (8%)		
Getting on/off chair	3 (14%)	2 (8%)			0 (0%)	0 (0%)			3 (8%)	2 (5%)		
Getting on/off toilet	0 (0%)	1 (4%)			0 (0%)	0 (0%)			0 (0%)	1 (3%)		
Going up/downstairs	0 (0%)	0 (0%)			0 (0%)	0 (0%)			0 (0%)	0 (0%)		
Walking between rooms	0 (0%)	0 (0%)			0 (0%)	0 (0%)			0 (0%)	0 (0%)		
Bending down or turning	1 (4%)	1 (4%)			0 (0%)	0 (0%)			1 (3%)	1 (3%)		
*Fall 2: Activity during fall*												
Housework	5 (23%)	6 (25%)	0.90	0.12	5 (36%)	6 (43%)	0.87	0.17	10 (28%)	12 (32%)	0.89	0.09
Getting out of bed	8 (36%)	8 (33%)			5 (36%)	5 (36%)			13 (36%)	13 (34%)		
Getting in/out of shower	4 (18%)	4 (17%)			1 (7%)	1 (7%)			5 (14%)	5 (13%)		
Getting on/off chair	4 (18%)	4 (17%)			1 (7%)	1 (7%)			5 (14%)	5 (13%)		
Getting on/off toilet	1 (4%)	2 (8%)			2 (14%)	1 (7%)			3 (8%)	3 (8%)		
Going up/downstairs	0 (0%)	0 (0%)			0 (0%)	0 (0%)			0 (0%)	0 (0%)		
Walking between rooms	0 (0%)	0 (0%)			0 (0%)	0 (0%)			0 (0%)	0 (0%)		
Bending down or turning	0 (0%)	0 (0%)			0 (0%)	0 (0%)			0 (0%)	0 (0%)		
*Fall 1: How fall occurred*												
Tripped	12 (55%)	14 (58%)	0.69	0.13	5 (36%)	5 (36%)	0.84	0.17	17 (47%)	19 (50%)	0.75	0.10
Slipped	6 (27%)	8 (33%)			8 (57%)	9 (64%)			14 (39%)	17 (45%)		
FoG	2 (9%)	2 (8%)			1 (7%)	0 (0%)			3 (8%)	2 (5%)		
Faint	2 (9%)	0 (0%)			0 (0%)	0 (0%)			0 (0%)	0 (0%)		
*Fall 2: How fall occurred*												
Tripped	12 (55%)	10 (42%)	0.82	0.16	7 (50%)	9 (64%)	0.75	0.24	19 (53%)	19 (50%)	0.80	0.13
Slipped	10 (45%)	13 (54%)			7 (50%)	5 (36%)			17 (47%)	18 (47%)		
FoG	0 (0%)	1 (4%)			0 (0%)	0 (0%)			0 (0%)	1 (3%)		
Faint	0 (0%)	0 (0%)			0 (0%)	0 (0%)			0 (0%)	0 (0%)		
*Fall 1: Direction of fall*												
Forwards	10 (45%)	12	0.76	0.16	8 (57%)	9 (64%)	0.88	0.25	18 (50%)	21 (55%)	0.80	0.13
Backwards	11 (50%)	10			6 (43%)	5 (36%)			17 (47%)	15 (39%)		
Side (Left)	1 (5%)	2			0 (0%)	0 (0%)			1 (3%)	2 (6%)		
Side (Right)	0 (0%)	0 (0%)			0 (0%)	0 (0%)			0 (0%)	0 (0%)		
*Fall 2: Direction of fall*												
Forwards	9 (41%)	9 (38%)	0.87	0.14	6 (43%)	6 (43%)	1.0	0.19	15 (42%)	15 (39%)	0.91	0.11
Backwards	5 (23%)	8 (33%)			5 (36%)	5 (36%)			10 (28%)	13 (34%)		
Side (Left)	8 (36%)	7 (29%)			3 (21%)	3 (21%)			11 (29%)	10 (26%)		
Side (Right)	0 (0%)	0 (0%)			0 (0%)	0 (0%)			0 (0%)	0 (0%)		
Most recent outdoor falls circumstances ^b^	*n* = 9	*n* = 6			*n* = 2	*n* = 2			*n* = 11	*n* = 8		
*Fall 1: Location of fall*												
Garden	3 (33%)	2 (33%)	0.67	0.44	1 (50%)	1 (50%)	1.0	0.71	4 (36%)	3 (38%)	0.76	0.24
Pavement	3 (33%)	4 (67%)			1 (50%)	1 (50%)			4 (36%)	5 (62%)		
Green space/beach	1 (11%)	0 (0%)			0 (0%)	0 (0%)			1 (9%)	0 (0%)		
Public transport	0 (0%)	0 (0%)			0 (0%)	0 (0%)			0 (0%)	0 (0%)		
Carpark	0 (0%)	0 (0%)			0 (0%)	0 (0%)			0 (0%)	0 (0%)		
Missing ^c^	2 (23%)	–			–	–			2 (18%)	–		
*Fall 2: Location of fall*												
Garden	2 (22%)	2 (33%)	0.86	0.29	1 (50%)	1 (50%)	1.0	0.71	3 (27%)	3 (38%)	0.89	0.25
Pavement	1 (11%)	2 (33%)			1 (50%)	1 (50%)			2 (18%)	3 (38%)		
Green space/beach	0 (0%)	0 (0%)			0 (0%)	0 (0%)			0 (0%)	0 (0%)		
Public transport	2 (22%)	1 (17%)			0 (0%)	0 (0%)			2 (18%)	1 (12%)		
Carpark	1 (11%)	1 (17%)			0 (0%)	0 (0%)			1 (9%)	1 (12%)		
Missing ^c^	3 (33%)	–			–	–			3 (27%)	–		
*Fall 1: Activity undertaken when fell*												
Gardening	2 (22%)	3 (50%)	0.75	0.29	1 (50%)	1 (50%)	–	–	3 (27%)	4 (50%)	0.82	0.27
Putting out rubbish	1 (11%)	1 (17%)			0 (0%)	0 (0%)			1 (9%)	1 (12%)		
Going up/downstairs	2 (22%)	1 (17%)			0 (0%)	0 (0%)			2 (18%)	1 (12%)		
Getting in/out of vehicle	0 (0%)	0 (0%)			0 (0%)	0 (0%)			0 (0%)	0 (0%)		
Walking	1 (11%)	1 (17%)			1 (50%)	1 (50%)			2 (18%)	2 (25%)		
Exercising	0 (0%)	0 (0%)			0 (0%)	0 (0%)			0 (0%)	0 (0%)		
Bending or turning	0 (0%)	0 (0%)			0 (0%)	0 (0%)			0 (0%)	0 (0%)		
Missing ^c^	3 (33%)	–			–	–			3 (27%)	–		
*Fall 2: Activity undertaken when fell*												
Gardening	3 (33%)	2 (33%)	0.89	0.33	1 (50%)	1 (50%)	1.0	0.71	4 (36%)	3 (38%)	0.91	0.28
Putting out rubbish	0 (0%)	1 (17%)			0 (0%)	0 (0%)			0 (0%)	1 (12%)		
Going up/downstairs	0 (0%)	0 (0%)			0 (0%)	0 (0%)			0 (0%)	0 (0%)		
Getting in/out of vehicle	2 (22%)	2 (33%)			0 (0%)	0 (0%)			2 (18%)	2 (25%)		
Walking	1 (11%)	1 (17%)			1 (50%)	1 (50%)			2 (18%)	2 (25%)		
Exercising	0 (0%)	0 (0%)			0 (0%)	0 (0%)			0 (0%)	0 (0%)		
Bending or turning	0 (0%)	0 (0%)			0 (0%)	0 (0%)			0 (0%)	0 (0%)		
Missing ^c^	–	–			–	–			3 (27%)	–		
*Fall 1: How fall occurred*												
Tripped	6 (66%)	6 (100%)	0.91	0.38	2 (100%)	1 (50%)	–	–	8 (73%)	7 (88%)	0.62	0.29
Slipped	1 (11%)	0 (0%)			0 (0%)	1 (50%)			1 (9%)	1 (12%)		
FoG	0 (0%)	0 (0%)			0 (0%)	0 (0%)			0 (0%)	0 (0%)		
Faint	0 (0%)	0 (0%)			0 (0%)	0 (0%)			0 (0%)	0 (0%)		
Missing ^c^	2 (22%)	–			–	–			2 (18%)	–		
*Fall 2: How falls occurred*												
Tripped	5 (56%)	5 (83%)	0.91	0.38	2 (100%)	2 (100%)	–	–	7 (64%)	7 (88%)	0.74	0.31
Slipped	2 (22%)	1 (17%)			0 (0%)	0 (0%)			2 (18%)	1 (12%)		
FoG	0 (0%)	0 (0%)			0 (0%)	0 (0%)			0 (0%)	0 (0%)		
Faint	0 (0%)	0 (0%)			0 (0%)	0 (0%)			0 (0%)	0 (0%)		
Missing ^c^	2 (22%)	–			–	–			2 (18%)	–		
*Fall 1: Direction of fall*												
Forwards	4 (44%)	3 (50%)	0.79	0.32	2 (100%)	2 (100%)	–	–	6 (55%)	5 (63%)	0.81	0.29
Backwards	1 (11%)	2 (33%)			0 (0%)	0 (0%)			1 (9%)	2 (25%)		
Side (Left)	1 (11%)	1 (17%)			0 (0%)	0 (0%)			1 (9%)	1 (12%)		
Side (Right)	0 (0%)	0 (0%)			0 (0%)	0 (0%)			0 (0%)	0 (0%)		
Missing ^c^	3 (33%)	–			–	–			3 (27%)	–		
*Fall 2: Direction of fall*												
Forwards	4 (44%)	4 (66%)			2 (100%)	1 (50%)	–	–	6 (55%)	5 (63%)	0.67	0.29
Backwards	3 (33%)	1 (17%)			0 (0%)	1 (50%)			3 (27%)	2 (25%)		
Side (Left)	1 (11%)	1 (17%)			0 (0%)	0 (0%)			1 (9%)	1 (12%)		
Side (Right)	1 (11%)	0 (0%)			0 (0%)	0 (0%)			1 (9%)	0 (0%)		

Values are presented as number and percentage (n [%]) for nominal, or median and interquartile range (IQR) for ordinal questions, with κ and SE of κ results for each individual question provided

*FoG* freezing of gait. Only data for the participants’ two most recent falls were included

^a^Percentages are based on the number of responses provided for indoor falls for the online version (*n* = 22 test, *n* = 24 retest), paper version (*n* = 14 for test and retest) and for both versions combined (*n* = 36 test, *n* = 38 retest)

^b^Percentages are based on the number of responses provided for outdoor falls for the online version (*n* = 9 test, *n* = 6 retest), paper version (*n* = 2 for test and retest) and for both versions combined (*n* = 11 test, *n* = 8 retest)

^c^Data was entered as missing if participants provided an answer of *“do not remember”,* or if they did not provide any response

**Table 3:** Responses and test-retest reliability for the near-falls circumstances questions, including the location and direction off the near-fall, the activity preceding the near-fall and how the near-fall occurred

Near-Falls Questions	Online Version (*n* = 30)				Paper Version (*n* = 16)				Combined Versions (*n* = 46)			
	Test	Retest	Test-retest reliability		Test	Retest	Test-retest reliability		Test	Retest	Test-retest reliability	
			Kappa	SE			Kappa	SE			Kappa	SE
Have you experienced a near-fall in the past 12-months? n (%)	30 (100%)	30 (100%)	–	–	16 (100%)	16 (100%)	–	–	46 (100%)	46 (100%)	–	–
Number of near-falls in the past 12-months; median (IQR)	3 (2–3)	3 (2–3)	0.44	0.12	3 (2–3)	2 (2–3)	0.58	0.17	3 (2–3)	2 (2–3)	0.51	0.11
Location of near-falls in the past 12-months; n (%)												
Indoor	4 (13%)	4 (13%)	1.00	0.14	1 (6%)	1 (6%)	1.00	0.31	5 (11%)	5 (11%)	1.00	0.12
Outdoor	6 (20%)	6 (20%)			1 (6%)	1 (6%)			7 (15%)	7 (15%)		
Both	20 (67%)	20 (67%)			14 (88%)	14 (88%)			34 (74%)	34 (74%)		
Number of indoor near-falls in the past 12-months; median (IQR)	3 (2–3)	2 (2–2)	0.44	0.12	2 (2–3)	2 (2–2)	0.53	0.16	2.5 (2–3)	2 (2–3)	0.47	0.09
Number of outdoor near-falls in the past 12-months; median (IQR)	3 (2–3)	3 (2–3)	0.56	0.14	3 (2–3)	3 (2.5–3)	0.56	0.16	3 (2–3)	3 (2–3)	0.56	0.10
Most recent near-falls circumstances; n (%)												
*Near fall 1: How the near-fall occurred?*												
Tripped	21 (70%)	20 (67%)	0.41	0.14	10 (63%)	9 (56%)	0.77	0.19	31 (67%)	29 (63%)	0.56	0.11
Slipped	7 (23%)	8 (27%)			4 (25%)	5 (31%)			11 (24%)	13 (28%)		
FoG	1 (3%)	1 (3%)			1 (6%)	1 (6%)			2 (4%)	2 (4%)		
Fainted	1 (3%)	1 (3%)			1 (6%)	1 (6%)			2 (4%)	2 (4%)		
*Near fall 2: How the near-fall occurred?*												
Tripped	13 (43%)	16 (53%)	0.66	0.14	7 (44%)	8 (50%)	0.33	0.22	20 (44%)	24 (52%)	0.56	0.12
Slipped	13 (43%)	12 (40%)			9 (56%)	7 (44%)			22 (47%)	19 (41%)		
FoG	4 (13%)	2 (7%)			0 (0%)	1 (6%)			4 (9%)	3 (7%)		
Fainted	0	0			0	0			0	0		
*Near fall 1: What activities you were doing?*												
Transitioning ^a^	16 (53%)	17 (57%)	0.59	0.15	11 (69%)	12 (75%)	0.75	0.21	27 (59%)	29 (64%)	0.65	0.12
Walking	7 (23%)	9 (30%)			2 (12%)	2 (12%)			9 (20%)	11 (24%)		
Turning/ Bending over	5 (17%)	2 (7%)			2 (12%)	1 (6%)			7 (15%)	3 (6%)		
Missing ^b^	2 (7%)	2 (7%)			1 (6%)	1 (6%)			3 (6%)	3 (6%)		
*Near fall 2: What activities you were doing?*												
Transitioning ^a^	7 (23%)	7 (23%)	0.78	0.18	5 (31%)	5 (31%)	0.79	0.26	12 (26%)	12 (26%)	0.79	0.14
Walking	9 (30%)	7 (23%)			2 (12%)	2 (12%)			11 (24%)	9 (20%)		
Turning/ Bending over	7 (23%)	4 (13%)			8 (50%)	3 (19%)			15 (33%)	7 (15%)		
Missing ^b^	7 (23%)	12 (40%)			1 (6%)	6 (38%)			8 (17%)	18 (39%)		
*Near fal1 l: Which direction did you nearly fall?*												
Forwards	14 (47%)	14 (47%)	0.51	0.14	9 (56%)	7 (44%)	0.48	0.17	23 (50%)	21 (46%)	0.54	0.11
Backwards	7 (23%)	12 (40%)			4 (25%)	6 (38%)			11 (24%)	18 (39%)		
Side (Left)	4 (13%)	3 (10%)			2 (13%)	2 (13%)			6 (13%)	5 (11%)		
Side (Right)	2 (7%)	1 (3%)			1 (6%)	1 (6%)			3 (6%)	2 (4%)		
Missing ^b^	3 (10%)	–			–	–			3 (6%)	–		
*Near fall 2: Which direction did you nearly fall?*												
Forwards	15 (50%)	13 (43%)	0.46	0.13	8 (50%)	7 (44%)	0.38	0.16	23 (50%)	20 (44%)	0.43	0.10
Backwards	11 (37%)	9 (30%)			7 (44%)	4 (24%)			18 (39%)	13 (28%)		
Side (Left)	1 (3%)	4 (13%)			0 (0%)	3 (19%)			1 (2%)	7 (15%)		
Side (Right)	2 (7%)	3 (10%)			1 (6%)	2 (13%)			3 (6%)	5 (11%)		
Missing ^b^	1 (3%)	1 (3%)			–	–			1 (2%)	1 (2%)		

Values are presented as number and percentage (n[%]) for nominal, or median and interquartile range (IQR) for ordinal questions, with κ and SE of κ results for each question provided

*FoG* freezing of gait. Only data for the participants’ two most recent falls were included

^a^Transitioning includes getting up from chair/bed/car

^b^Data was entered as missing if participants provided an answer of *“do not remember”,* or if they did not provide any response

**Table 4:** Responses and test-retest reliability for the injurious falls circumstances questions, including the number and location of the injurious fall, type of injury sustained and medical treatment sought

Injurious Falls Questions	Online Version (*n* = 30)				Paper Version (*n* = 16)				Combined Versions (*n* = 46)			
	Test	Retest	Test-retest reliability		Test	Retest	Test-retest reliability		Test	Retest	Test-retest reliability	
			Kappa	SE			Kappa	SE			Kappa	SE
Have you suffered an injurious fall in the past 12-months? n (%)	9 (30%)	9 (30%)	0.84	0.18	6 (38%)	6 (38%)	1.0	0.25	15 (33%)	15 (33%)	0.90	0.15
Number of injurious indoor falls; median (IQR)	2 (2–2)	2 (1–2)	0.56	0.26	2 (2–2)	2 (1–2)	0.63	0.26	2 (2–2)	2 (1–2)	0.59	0.19
Number of injurious outdoor falls; median (IQR)	1 (1–1)	1 (1–1)	–	–	1 (1–1)	1 (1–1)	–	–	1 (1–1)	1 (1–1)	–	–
Of the injurious indoor falls in the past 12-months, how many times did you see a healthcare professional? n (%) ^a^												
1–2	3 (33%)	5 (56%)	0.63	0.27	3 (50%)	4 (66%)	0.67	0.39	6 (40%)	9 (60%)	0.65	0.21
3–4	3 (33%)	1 (11%)			3 (50%)	2 (33%)			6 (40%)	3 (20%)		
5–6	1 (11%)	1 (11%)			0 (0%)	0 (0%)			1 (7%)	1 (7%)		
7–8	0 (0%)	0 (0%)			0 (0%)	0 (0%)			0 (0%)	0 (0%)		
≥ 9	0 (0%)	0 (0%)			0 (0%)	0 (0%)			0 (0%)	0 (0%)		
Missing ^b^	2 (23%)	2 (23%)			–	–			2 (13%)	2 (13%)		
Of the injurious outdoor falls in the past 12-months, how many times did you see a healthcare professional? n (%) ^a^												
1–2	0 (0%)	0 (0%)	–	–	–	–	–	–	0 (0%)	0 (0%)	–	–
3–4	1 (11%)	0 (0%)			–	–			1 (7%)	0 (0%)		
5–6	0 (0%)	0 (0%)			–	–			0 (0%)	0 (0%)		
7–8	0 (0%)	0 (0%)			–	–			0 (0%)	0 (0%)		
≥ 9	0 (0%)	0 (0%)			–	–			0 (0%)	0 (0%)		
Missing ^b^	8 (89%)	9 (100%)			6 (100%)	6 (100%)			14 (93%)	15 (100%)		
Most recent injurious falls												
*Fall 1: Did you suffer from any of the following injuries? n (%)* ^*c*^	*n* = 14	*n* = 14			*n* = 7	*n* = 7			*n* = 21	*n* = 21		
Cuts/abrasions	0 (0%)	0 (0%)	0.61	0.18	0 (0%)	0 (0%)	0.48	0.27	0 (0%)	0 (0%)	0.57	0.15
Bruises	3 (21%)	3 (21%)			3 (43%)	4 (57%)			6 (30%)	7 (35%)		
Back Pain	7 (50%)	6 (43%)			2 (29%)	2 (29%)			9 (45%)	8 (40%)		
Muscle strain	3 (21%)	4 (29%)			1 (14%)	0 (0%)			4 (20%)	4 (20%)		
Joint dislocation	0 (0%)	0 (0%)			0 (0%)	0 (0%)			0 (0%)	0 (0%)		
Fracture	1 (7%)	1 (7%)			1 (14%)	1 (14%)			2 (5%)	2 (5%)		
*Fall 2: Did you suffer from any of the following injuries? n (%)* ^*c*^	*n* = 6	*n* = 6			*n* = 3	*n* = 3			*n* = 9	*n* = 9		
Cuts/abrasions	2 (33%)	2 (33%)	0.54	0.31	0 (0%)	0 (0%)	1.0	0.58	2 (22%)	2 (22%)	0.75	0.23
Bruises	3 (50%)	3 (50%)			2 (67%)	2 (67%)			5 (56%)	5 (56%)		
Back Pain	1 (17%)	1 (17%)			0 (0%)	0 (0%)			1 (11%)	1 (11%)		
Muscle strain	0 (0%)	0 (0%)			1 (33%)	1 (33%)			1 (11%)	1 (11%)		
Joint dislocation	0 (0%)	0 (0%)			0 (0%)	0 (0%)			0 (0%)	0 (0%)		
Fracture	0 (0%)	0 (0%)			0 (0%)	0 (0%)			0 (0%)	0 (0%)		
Did you seek any medical treatment for the injuries? n (%) ^*c*^	*n* = 17	*n* = 15			*n* = 8	*n* = 6			*n* = 25	*n* = 21		
Hospitalisation	8 (47%)	7 (47%)	0.86	0.22	3 (38%)	3 (50%)	0.96	0.33	11 (44%)	10 (48%)	0.89	0.18
Stitches	1 (6%)	0 (0%)			1 (12%)	0 (0%)			2 (8%)	0 (0%)		
Dressing of injury	0 (0%)	0 (0%)			0 (0%)	0 (0%)			0 (0%)	0 (0%)		
X-ray/CT scan/MRI	4 (23%)	3 (20%)			3 (38%)	2 (33%)			7 (28%)	5 (24%)		
Physiotherapy/ occupational therapy	4 (23%)	5 (33%)			1 (12%)	1 (17%)			5 (20%)	6 (29%)		

^a^Percentages are based on the number of people who responded yes to suffering an injurious fall during test and retest for online (*n* = 9), paper (*n* = 6) and combined (*n* = 15)

^b^Data was entered as missing if participants provided an answer of *“do not remember”,* or if they did not provide any response

^c^For the questions concerning injuries and medical treatment following the most recent falls events, as participants could respond with multiple answers (e.g., bruises and back pain), percentages are based on the total number of responses provided for that question

The overall average κ score for the fall’s circumstances (location, activity, how, and direction) domain based on the pooled questionnaire results was 0.79, which represented moderate agreement. As shown in Table 2, the test-retest agreement was generally moderate to strong for each question related to falls. The question concerning the number of falls in the past 12 months demonstrated weak agreement (*κ* = 0.41), but the questions concerning the location of falls (*κ* = 0.89) and the number of indoor (*κ* = 0.86) and outdoor (*κ* = 0.75) falls all demonstrated moderate to strong agreement. For the most recent indoor and outdoor fall, there was moderate to strong agreement for the questions related to the location (indoor fall 1 *κ* = 0.74; outdoor fall 1 *κ* = 0.76), direction (indoor fall 1 *κ* = 0.80; outdoor fall 1 *κ* = 0.81), activity (indoor fall 1 *κ* = 0.70; outdoor fall 1 *κ* = 0.82;), and cause (indoor fall 1 *κ* = 0.75; outdoor fall 1 *κ* = 0.62;). Similar results were observed for the second most recent indoor and outdoor fall questions regarding the circumstances (location, activity, how and direction) (Table 2).

All participants reported having a near-fall in the previous 12-months, and therefore κ values were not presented for the first question. The overall κ score for the near-fall circumstances (location, activity, how, and direction) domain for the pooled questionnaire results was 0.60, which represented moderate agreement. Questions assessing the number of near-falls in the previous 12-months demonstrated weak agreement (*κ* = 0.51), while questions concerning the location of near-falls demonstrated almost perfect agreement (*κ* = 1.0) (Table 3). In contrast, there was a weak agreement for questions related to the number of indoor (*κ* = 0.47) and outdoor (*κ* = 0.56) near-falls, while the direction (near-fall 1 *κ* = 0.54), activity (near-fall 1 *κ* = 0.65) cause (near-fall 1 *κ* = 0.56) of near-falls questions all demonstrated between weak to moderate agreement (Table 3). Once more, similar results were observed for the circumstances (location, activity, how and direction) of the second most recent near-falls questions (Table 3).

The overall average κ score for the injurious falls domain based on the pooled questionnaire results was 0.73, which represented moderate agreement. As shown in Table 4, the test-retest agreement was moderate to strong for the individual questions concerning whether participants experienced an injurious fall in the past 12-months (*κ* = 0.90), the number of injurious falls (indoor *κ* = 0.59), number of visits to a healthcare professional (indoor *κ* = 0.65), injuries sustained as a result of an injurious fall (fall 1 *κ* = 0.57; fall 2 *κ* = 0.75) and medical treatment sought for any injurious fall (*κ* = 0.89). Due to only one person in this cohort experiencing a fracture, the κ values were not provided for fracture-related questions. Similarly, only one person reported experiencing an injurious outdoor fall, and so the κ values for these questions were also not provided.

## Discussion

To our knowledge, the PDF-Q used in this study is the first falls-specific questionnaire to be developed and tested for reliability among older adults with PD. The main finding from this study was that the pooled results from the online and paper modes for the PDF-Q collectively demonstrated moderate to strong agreement (reliability) scores for most questions related to the circumstances and consequences of falls and near-falls, though there was a weaker agreement for the number of falls and near-falls questions. These findings indicate that the PDF-Q represents a reliable tool to capture the circumstances and consequences of falls and near-falls in older adults with PD, yet the recall length (previous 12-months) may be too long to accurately remember the number of falls and near-falls sustained.

Since there is no previous data on the reliability of PD-specific falls-related questionnaires, it is difficult to make direct comparisons with our test-retest results. In non-PD older adults, there is data on the test-retest reliabilities of both the Carefall Triage Instrument [50] and the Falls Efficacy Scale-International (FES-I) [51]. The Carefall Triage Instrument demonstrated minimal to strong test-retest reliability for self-rated questions assessing falls risk among 27 older adults aged over 65 years, while the FES-I has demonstrated strong test-retest reliability for questions related to concerns about falling among 16 older adults aged between 60 and 95 years. However, due to the diverse statistical approaches implemented (e.g., the FES-I was analysed using Cronbach’s α and intraclass correlation coefficients) and the differences between domain designs of the The Carefall Triage instrument and the FES-I compared to the PDF-Q, it is difficult to directly compare the reliability results of each questionnaire concerning outcomes such as the number, circumstances and consequences of falls. Furthermore, the PDF-Q developed in this study is the first of its kind to establish the test-retest reliability of questions capturing near-falls information among older adults with PD. To our knowledge, no studies have evaluated the test-retest reliability of a near-falls questionnaire, and our pooled κ results for near-falls indicates that there was generally a moderate level of agreement for these questions.

As a comparison between the pooled PDF-Q domains for falls and near-falls, there are some potential explanations for why the near-falls circumstances domain (*κ* = 0.60) demonstrated relatively weaker reliability than the circumstances of the falls domain (*κ* = 0.79), although both domains still demonstrated moderate reliability. Firstly, near-fall events may be a less traumatic experience compared to an actual fall, particularly if the individual experiences an injury as a result of the fall [52, 53]. Indeed, we established a strong agreement for the question concerning whether participants received medical treatment for injury following a fall (*n* = 21, *κ* = 0.89). Thus, it may be that older adults with PD are more likely to recall the circumstances of events preceding a fall resulting in injury, compared to near-falls where injuries are less likely. Jayasinghe et al., [54] showed that 27% of older adult (non-PD) fallers (from a sample of 100) had post-traumatic stress as a result of the fall, and 78% reported ‘feeling emotionally upset when reminded of the fall’. It is also possible the 12-month recall period was too long for the participants to recall the circumstances of near-falls, especially given they are typically not traumatic events. Certainly, there is evidence that older adults may have difficulty recalling non-traumatic near-fall events beyond 3-months [53, 55], which may be worse in older adults with PD whom often have deficits in working memory, or mild cognitive impairments [53, 56]. For instance, it has been reported that 45% of older adults with PD had memory deficits compared to healthy age-matched controls [57]. However, cognitive deficits are unlikely to explain the disproportionate near-falls and falls reliability results in this study as participants with MCI were excluded. Finally, it may have been difficult for the older adults in this study to differentiate near-falls events from stumbles/trips, where a fall was not going to occur even without the support of a wall or railing etc. [17]. In the PDF-Q we adopted the definition of a near-fall from Maidan et al., [58] and Lindholm et al., [34] which was defined as *“any incident where you may have fallen but were able to catch yourself and did not come to rest on the ground”*. Although this definition may be open to interpretation, we were still able to demonstrate mostly moderate levels of agreement for each question within the near-falls domain. As such, despite the relatively weaker reliability scores compared to the circumstances of the falls domain, we believe that the PDF-Q represents a reliable tool to capture near-falls information in older adults with PD.

The reliability results for the questions concerning the number of falls and near-falls showed relatively weaker agreement compared to the circumstances and location of falls and near-falls questions. Previous studies have indicated that recall length using retrospective surveys should focus on shorter periods (e.g., less than 3-months) to reduce memory bias and improve the precision of the responses [46, 50]. As such, this may explain the poorer reliability scores for these questions which focussed on recalling the number of falls and near-falls events over the previous 12-months. However, further research is needed investigating the reliability of these questions over shorter recall lengths to confirm this contention.

The pooled results for the injurious falls domain demonstrated moderate to strong agreement for each question within the PDF-Q. To our knowledge, no studies have reported test-retest reliability for injurious falls among older adults with PD. In this study, data from 15 participants were used for the test-retest analysis on the number and circumstances of injurious falls in the past 12-months. As such, the reliability results for the injurious falls questions must be interpreted with caution, as it has been reported that at least 25 participants are required for acceptable test-retest reliability analysis [38]. Nevertheless, the reliability results indicating a strong agreement may be generalisable for larger cohorts of older adults with PD who suffer injurious falls. For example, our participants, on average, had similar characteristics, and reported a comparable injurious falls rate (33%), to previous epidemiology studies investigating injurious falls among older adults with PD [19, 22, 59]. Still, further research should consolidate our reliability results among a larger sample (*n* > 25) of individuals with PD who have suffered an injurious fall(s) to strengthen these findings.

A strength of this study was the step-wise development of the PDF-Q, which included a literature search and expert consensus regarding the questions and domains of the instrument. However, there are several limitations associated with this study that must be acknowledged. Firstly, we experienced difficulties in recruiting older adults with PD to complete the paper-based version of the PDF-Q, and due to this small sample size (*n* = 16) we were unable to directly compare the test-retest reliability findings with the online version. Nevertheless, it is worth noting that for many of the individual questions there was a similar level of agreement (reliability) between the online and paper-based version. Consistent with this observation, several previous studies have shown comparable responses (and similar reliabilities) between paper and online questionnaires among healthy populations [40, 60–62]. Additionally, one study compared online and paper versions of the 39-item self-report Parkinson’s disease questionnaire (PDQ-39) among 44 older adults with PD, and found little differences between modes regarding responsiveness and data precision [63]. Second, a four-week wash-out period between testing and retesting days was adopted in this study. Although this is standard practice for test-retest reliability analysis, we did not capture any information about falls or near-falls events within these 4 weeks (e.g., via falls diaries). Given that older adults with PD are a high-risk group for recurrent falls [9, 64], it cannot be determined if the reported circumstances surrounding falls and near-falls between the test and retest periods are accurately assessing the same ‘*most recent’* fall/near-fall event following the four-week washout period. However, we postulate that given the acceptable levels of agreement scores for the majority of questions, many of the most recent falls/near-falls remained the same even after 4 weeks. Third, we acknowledge that with the falls and near-falls domains some descriptive questions presented lower agreement scores compared to others, which may have been the result of inadequate instruction; particularly concerning the order of the most recent events. However, given the high number of “don’t remember” responses from participants for falls/near-falls 3 and 4 compared to falls/near-falls 1 and 2, we suspect that participants interpreted fall/near-fall 1 as being their most recent event and fall/near-fall 2 as their second most recent, and so on. Indeed, it may be that older adults with PD find it difficult to recall falls/near-falls events beyond the two most recent events [52, 53]. Researchers and clinicians using the PDF-Q should, therefore, consider reducing the recall period for more reliable responses.

## Conclusions

In conclusion, the new PDF-Q developed in this study was found to be reliable for capturing the circumstances and consequences of recent falls and near-falls in older adults with PD, but showed weaker agreement for the questions concerning the number of falls and near-falls in the previous 12-months. It is possible that the recall period was too long for participants to accurately remember the number of falls and near-falls events experienced, though more research is needed to investigate the reliability of these questions using shorter recall lengths to confirm this. There was also a comparatively weaker agreement between the near-falls circumstances domain and the circumstances of the falls domain of this questionnaire. This is possibly due to older adults with PD being less able to precisely recall the events surrounding near-falls, which are generally less traumatic compared to falls. However, further exploration is needed comparing the precision of recalling the events surrounding near-falls to falls among older adults with PD to verify our contention. The step-wise design of the PDF-Q was sufficient in establishing content validity of the instrument. Despite this, future studies should aim to establish criterion and construct validities of the PDF-Q. Overall, this is the first attempt of any kind to develop and demonstrate test-retest reliability of a PD-specific falls questionnaire, which will serve as a valuable tool for accurately capturing falls, near-falls and injurious falls information from older adults with PD.

## Supplementary Information

**Additional file 1.**

## Data Availability

The datasets analysed during the current study are available from the corresponding author on reasonable request.

## Abbreviations

PD: Parkinson’s disease
PDF-Q: Parkinson’s diease falls questionnaire
PDQ-39: 39-item self-report Parkinson’s disease questionnaire
FRQ: Falls Risk Questionnaire
FES-I: Falls Efficacy Scale-International
SF-36: Short Form 36 Health Survey
