# Supplementary material for: Development of a Parkinson’s disease specific falls questionnaire
Source: BMC Geriatr. 2021 Oct 30;21:614. doi: 10.1186/s12877-021-02555-6 (PMC8557480; doi:10.1186/s12877-021-02555-6)
Supplement: Supplementary file 1 — Additional file 1. [file 12877_2021_2555_MOESM1_ESM.docx]

**Parkinson Disease Falls Questionnaire (PDF-Q)**

**In this questionnaire, we will ask you questions about your personal history, Parkinson’s disease diagnosis and history of falls circumstances and consequences. Please allow approximately 20 minutes to complete this questionnaire. All responses will remain confidential.**

**Section 1: Consent**

1. **Do you give your consent for the information in this questionnaire to be used for research all information will be identified and confidential? *(please tick the box)***
2. Yes I give consent ☐
3. **Please indicated who completed the questionnaire for you?**
4. I completed the questionnaire myself ☐
5. My carer completed the questionnaire for me ☐

If a carer completed the questionnaire, please indicate the relation to you (e.g. wife / husband / daughter etc) ________________

**Section 2: Demographics and Disease Status**

The questions in this section will ask about your physical characteristics and your Parkinson’s disease history. Please answer these questions to the best of your knowledge.

1. **What is your date of birth?**

__ __/ __ __ / __ __ __ __

1. **How old are you?**

_______ Yrs. _______ Months

1. **What is your self-reported Height?**

_______ cm or _______ ft. _____ inches

1. **What is your self-reported Weight?**

_______ Kg or _______ stone _____ lbs

1. **Have you been medically diagnosed with Parkinson’s disease?**

Yes ☐ No ☐

1. **How many years have you been suffering with Parkinson’s disease?**

_______ Yrs. ______ Months

1. **Are you currently taking medication to manage your Parkinson’s Disease?**

Yes ☐ No ☐

**Section 3: Falls History**

This section we will ask you questions in regards to any falls or near falls you may have had in the past 12 month. Please answers these questions to the best of your knowledge.

1. **Thinking of the last 12 months have you experienced any of the following?**
2. A near fall *(any incident where you may have fallen, but didn’t come to rest on the ground)* Yes ☐ No ☐
3. A Fall (*an incident that resulted in your unintentionally coming to rest on the ground)*

Yes ☐ No ☐

**If you answered no to both part a and b above, please do not proceed to the next section and end the questionnaire here.**

**Section 4: Circumstance and Consequences of Near Falls**

In this section we would like you to think about only the **NEAR FALLS** you have experienced in the past 12 months. ***Remember a near fall is any incident where you may have fallen but were able to catch yourself and did not come to rest on the ground?***

1. Thinking of the **past 12 months** how many times in total can you recall experiencing a **near fall?**

**Total number of Near Falls:**  _________ **None**  ☐ ***(Please proceed to Section 5)***

1. Thinking of your **Near Falls** over the **past 12 month**, in which location did the near falls occur? *Please tick below if the nears falls occurred indoors or outdoors and the total number of near falls in this location*
2. Indoors (in your home or another building etc) ☐ ___________ Total
3. Outdoors (in your yard, public transport, on a path etc) ☐ ___________ Total
4. Thinking of the **four most recent Near Falls** you have experienced in the **past 12 months** please indicate for each how the near fall occurred? *(please tick the box)*

| **Reason for Near Fall** | **Near Fall 1** | **Near Fall 2** | **Near Fall 3** | **Near Fall 4** |
| --- | --- | --- | --- | --- |
| I tripped |  |  |  |  |
| I slipped |  |  |  |  |
| I lost my balance |  |  |  |  |
| I felt faint/dizzy/unwell |  |  |  |  |
| Freezing of Gait |  |  |  |  |
| Other (please specify) |  |  |  |  |
| I do not remember |  |  |  |  |

1. Thinking of the **four most recent Near falls** you have experienced in the **past 12 months** please indicate for each what activities you were doing? *(please tick the box)*

| **Activity** | **Near Fall 1** | **Near Fall 2** | **Near Fall 3** | **Near Fall 4** |
| --- | --- | --- | --- | --- |
| Transitioning (ie from a chair/ bed / car) |  |  |  |  |
| Walking (outside or between rooms) |  |  |  |  |
| Turning or bending |  |  |  |  |
| Other (please specify) |  |  |  |  |
| I do not remember |  |  |  |  |

1. Thinking of your four most recent Near Falls you have had in the **past 12 months** please indicate which direction you nearly fell?

| **Direction** | **Near Fall 1** | **Near Fall 2** | **Near Fall 3** | **Near Fall 4** |
| --- | --- | --- | --- | --- |
| Forwards |  |  |  |  |
| Backwards |  |  |  |  |
| Sideways to my left side |  |  |  |  |
| Sideways to my right side |  |  |  |  |
| I do not remember |  |  |  |  |

**Section 5: Circumstance and Consequences of falls**

In this section we would like you to think about only the **FALLS** you have experienced in the past 12 months. ***REMEMBER a fall is:*** *when you unintentionally come to rest on the ground or some lower level from where you were standing, and not as a result of freezing, epileptic seizure or overwhelming external force. So for example, someone accidently bumps into you and you fall over, this would not be classed as a fall.*

1. Thinking of the past 12 months how many times in total have you experienced a fall

**Total number of Falls:**  _________ **None**  ☐ ***(Please proceed to end)***

1. Thinking of all the **falls** you have experienced in the **past 12 month**, in which location did the falls occur? *Please tick below if the falls occurred indoors or outdoors and the total number of falls in this location*

a. Indoors (in your home or another building etc) ☐ ___________ Total

b. Outdoors (in your yard, public transport, on a path etc) ☐ ___________ Total

1. Thinking specifically about the **INDOOR FALLS** you experienced **over the past 12 months** please indicate for your **four most recent falls** in which indoor location each fall occurred?

I did not have an indoor fall ☐ *(Please proceed to question 7 in this section)*

| **Location** | **Fall 1** | **Fall 2** | **Fall 3** | **Fall 4** |
| --- | --- | --- | --- | --- |
| In your kitchen / dining room |  |  |  |  |
| In your hallway / lobby / doorway |  |  |  |  |
| In your living / family room |  |  |  |  |
| In your bedroom |  |  |  |  |
| In your bathroom/ shower room/ toilet |  |  |  |  |
| On your stairs in the home |  |  |  |  |
| In another person’s home or building i.e shopping centre/ grocery store/ theatre etc |  |  |  |  |
| Other indoor location *(Please specify for each fall)* |  |  |  |  |
| I do not remember |  |  |  |  |

1. Thinking specifically about the **INDOOR FALLS** you experienced over the **past 12 months** please indicate for your **four most recent falls** what activity you doing when you fell?

| **Activity** | **Fall 1** | **Fall 2** | **Fall 3** | **Fall 4** |
| --- | --- | --- | --- | --- |
| General house work (cleaning/making dinner etc) |  |  |  |  |
| Getting out of bed |  |  |  |  |
| Getting in or out of the shower |  |  |  |  |
| Getting on or off of a chair |  |  |  |  |
| Getting on or off the toilet |  |  |  |  |
| Going up or down the stairs |  |  |  |  |
| Walking between rooms |  |  |  |  |
| Bending down or turning |  |  |  |  |
| Other indoor activity (Please specify for each fall) |  |  |  |  |
| I do not remember |  |  |  |  |

1. Thinking specifically about the **INDOOR FALLS** you experienced over the **past 12 months** please indicate for your **four most recent falls** how you fell?

| **Reason for falling** | **Fall 1** | **Fall 2** | **Fall 3** | **Fall 4** |
| --- | --- | --- | --- | --- |
| I tripped |  |  |  |  |
| I slipped |  |  |  |  |
| I lost my balance |  |  |  |  |
| I felt faint/dizzy/unwell |  |  |  |  |
| other |  |  |  |  |
| I do not remember |  |  |  |  |

1. Thinking specifically about the **INDOOR FALLS** you experienced over the **past 12 months** please indicate for your **four most recent falls** which direction you fall?

| **Direction of fall** | **Fall 1** | **Fall 2** | **Fall 3** | **Fall 4** |
| --- | --- | --- | --- | --- |
| Forwards |  |  |  |  |
| Backwards |  |  |  |  |
| Sideways to my **left** side |  |  |  |  |
| Sideways to my **right** side |  |  |  |  |
| I do not remember |  |  |  |  |

1. Thinking specifically about the **OUTDOOR FALLS** you experienced over the **past 12 months** please indicate for your **four most recent falls** in which outdoor location each fall occurred?

I did not have an outdoor fall ☐ *(Please proceed to Section 6 Injurious falls)*

| **Location** | **Fall 1** | **Fall 2** | **Fall 3** | **Fall 4** |
| --- | --- | --- | --- | --- |
| In the yard/ garden/ garage |  |  |  |  |
| On the pavement/ road/ public foot path |  |  |  |  |
| In a public park/ nature park / green space / beach |  |  |  |  |
| On public transport (bus/ train/ tram/ cruise ship) |  |  |  |  |
| In a carpark |  |  |  |  |
| Other outdoor location *(Please specify for each fall)* |  |  |  |  |
| I do not remember |  |  |  |  |

1. Thinking specifically about the **OUTDOOR FALLS** you experienced over the **past 12 months** please indicate for your **four most recent falls** what activity you doing when you fell?

| **Activity** | **Fall 1** | **Fall 2** | **Fall 3** | **Fall 4** |
| --- | --- | --- | --- | --- |
| Gardening/ yard work / lawn care |  |  |  |  |
| Putting out the rubbish or dust bins |  |  |  |  |
| Going up or down steps |  |  |  |  |
| Getting in /out or off of a vehicle (bus/ car/ bike) |  |  |  |  |
| Walking |  |  |  |  |
| Exercising |  |  |  |  |
| Bending down or turning |  |  |  |  |
| Other outdoor activity *(Please specify for each fall)* |  |  |  |  |
| I do not remember |  |  |  |  |

1. Thinking specifically about the **OUTDOOR FALLS** you experienced over the **past 12 months** please indicate for **your four most recent falls** how you fell?

| **Reason for falling** | **Fall 1** | **Fall 2** | **Fall 3** | **Fall 4** |
| --- | --- | --- | --- | --- |
| I tripped |  |  |  |  |
| I slipped |  |  |  |  |
| I lost my balance |  |  |  |  |
| I felt faint/dizzy/unwell |  |  |  |  |
| Freezing of Gait |  |  |  |  |
| Other *(please specify for each fall)* |  |  |  |  |
| I do not remember |  |  |  |  |

1. Thinking specifically about the **OUTDOOR FALLS** you experienced over the **past 12 months** please indicate for your **four most recent falls** which direction you fall?

| **Direction of fall** | **Fall 1** | **Fall 2** | **Fall 3** | **Fall 4** |
| --- | --- | --- | --- | --- |
| Forwards |  |  |  |  |
| Backwards |  |  |  |  |
| Sideways to my left side |  |  |  |  |
| Sideways to my right side |  |  |  |  |
| I do not remember |  |  |  |  |

**Section 6: Injurious Falls**

In this section we would like you to think about the injuries you have experience due to **FALLING** in the **past 12 months.**

1. Thinking about **ALL the falls** (indoors and outdoors) you have experienced over the **past 12 months** have any of these falls resulted in you sustaining an injury (including cuts, bruising sprains etc)?

Yes ☐ No ☐ *(please end the questionnaire here)*

1. Thinking of **all the falls** you have experienced in the **past 12 month**, how many in total have resulted in an **injury**? Please tick if the injurious fall occurred indoors or outdoors and the total number of injurious falls.
2. Indoors (in your home or another building etc) ☐ ___________ Total

b. Outdoors (in your yard, public transport, on a path etc) ☐ ___________ Total

1. Thinking of all the **INJURIOUS falls** you have experienced in the **past 12 month** on average how many times did you make an appointment to see a medical / health care professional? (e.g. physiotherapist, doctor, exercise physiologist etc.)

1-2 times ☐ 3-4 times ☐ 4-5 times ☐

5-6 times ☐ 7 or more ☐ None ☐

1. Thinking of all the **INJURIOUS falls** you have experienced in the **past 12 month** what injuries have you sustained due to the falls please tick the injury and the number of falls in which this injury occurred

| **Type of injury** | **Did the injury occur (if yes please tick)** | **Total number of falls** |
| --- | --- | --- |
| Cut/ Grazes / Abrasions |  |  |
| Bruises |  |  |
| Back Pain |  |  |
| Muscle Strains /Sprains |  |  |
| Other (please specify) |  |  |

1. Thinking of all the **INJURIOUS falls** you have experienced in the **past 12 month** and the injuries you have sustained please indicate what **treatment** you required because of fall, the number of **times on average you received the treatment** and the **number of falls in total** you required the treatment.

I did not require treatment ☐ *(Please proceed to question 6)*

| Treatment | I received the follow treatment (please tick each that apply) | Times receiving the treatment on average for each fall | Number of falls requiring the treatment |
| --- | --- | --- | --- |
| Hospitalisation |  | ____________  Average number of days |  |
| Stitches |  |  |  |
| Dressing of injury |  |  |  |
| X-Ray/ CT /MRI |  |  |  |
| Physiotherapy |  |  |  |
| Other (please specify) |  |  |  |

1. Thinking of all the **INJURIOUS falls** you have experienced in the **past 12 month** please indicate how many falls resulted in a **bone fracture**
2. Total falls resulting in a bone fracture ___________
3. None ☐ *(please end the questionnaire here)*
4. Thinking of the falls you have experienced in the **past 12 month** that have resulted in a **bone fracture** please indicate which body site was fractured/broken and the number of times it was broken due to the fall.

| Site of fractures | Number of falls | Site of fractures | Number of falls |
| --- | --- | --- | --- |
| Wrist/ forearm (ulna or radius) |  | Toes |  |
| Upper arm (Humerus) |  | Ankle |  |
| Collar bone (Clavicle) |  | Lower leg (tibia/ fibula) |  |
| Shoulder Blade (Scapula) |  | Upper leg (femur) |  |
| Head or Face (skull) |  | Hip (femur Head) |  |
| Spine (lumbar/cervical/ thoracic) |  | Pelvis |  |
| Ribs |  |  |  |

1. Thinking of the falls you have experienced in the **past 12 month** that have resulted in a **bone fracture** please indicate what treatment you received. Please

| Treatment | Treatment (please tick) | Number of fractures | Times/ days receiving treatment on average |
| --- | --- | --- | --- |
| Hospitalisation |  |  |  |
| Emergency room treatment |  |  |  |
| Bed rest at home |  |  |  |
| Immobilization in a splint or cast |  |  |  |
| Strapping or sling |  |  |  |
| Surgical fixation |  |  |  |
| X-ray |  |  |  |

**Thank you for completing this Questionnaire!**
